# Paleoparasitological evidence of pinworm (*Enterobius vermicularis*) infection in a female adolescent residing in ancient Tehran (Iran) 7000 years ago

**DOI:** 10.1186/s13071-016-1322-y

**Published:** 2016-01-22

**Authors:** Niloofar Paknazhad, Gholamreza Mowlavi, Jean Dupouy Camet, Mohammad Esmaeili Jelodar, Iraj Mobedi, Mahsasadat Makki, Eshrat Beigom Kia, Mostafa Rezaeian, Mehdi Mohebali, Siamak Sarlak, Faezeh Najafi

**Affiliations:** Department of Parasitology and Mycology, School of Public Health, Tehran University of Medical Sciences, Tehran, Iran; Center for Research of Endemic Parasites of Iran (CREPI), Tehran University of Medical Sciences, Tehran, Iran; Service de Parasitologie-Mycologie, Hôpital Cochin Assistance Publique Hôpitaux de Paris, Université Paris Descartes, 27 Faubourg St Jacques, 75014 Paris, France; Department of Archaeology Faculty of Letters and Humanities University of Tehran, Tehran, Iran; Member of Iranian Center for Archaeological Research, Siye Tir Street, Imam Khomeini Avenue, Tehran, Iran

**Keywords:** Paleoparasitology, *Enterobius vermicularis*, Tehran, Iran

## Abstract

**Background:**

The Molavi street archeological site south of Tehran accidentally provided a unique opportunity for paleoparasitological studies in Iran. A female skeleton was unearthed and evaluated to be 7000 years old. Soil samples were collected around the pelvic and sacrum bones.

**Findings:**

Careful microscopic investigation of rehydrated soil samples revealed the presence of one *Enterobius vermicularis* egg attached to the skeleton sacral region.

**Conclusion:**

The present finding likely represents the oldest evidence of a human pinworm infection in Asia.

## Findings

### Background

Biological remains that are excavated from archeological sites are the main source of parasites that existed in ancient times and paleoparasitologists, can take advantage of organic remains to identify ancient parasites of humans and animals. Therefore, coprolites, burial soil samples and occasionally suspected parasitic objects [1] are always valuable material to paleoparasitologists. Excavation of a female skeleton at the “Molavi street” archeological site, has not only pushed back the presence of humans living in Tehran to seven thousand years ago, but has also demonstrated the oldest occurrence of a pinworm infection on the Asian continent. Former studies indicated that *Enterobius vermicularis* could be considered as the most ancient parasite of our human ancestors [2]. This exclusive obligate parasite of humans, directly transmitted from person to person, has a worldwide distribution and is very common in temperate and cool climate regions [3]. Evidence of pinworm infections in ancient populations has been demonstrated at different archeological sites on the American continent, Europe and Asia [4]. In Iran, eggs of *E. vermicularis* and *Oxyuris equi* have been found in soil samples collected from the Chehrabad salt mine archeological site and dated back to between 1500–2500 years BC [5]. Moreover, eggs of *Syphacia obvelata* a common oxyurid worm of mice and rats have also been diagnosed in rodent coprolites excavated from the same archeological site [6].

## Methods

**Fig. 1:**
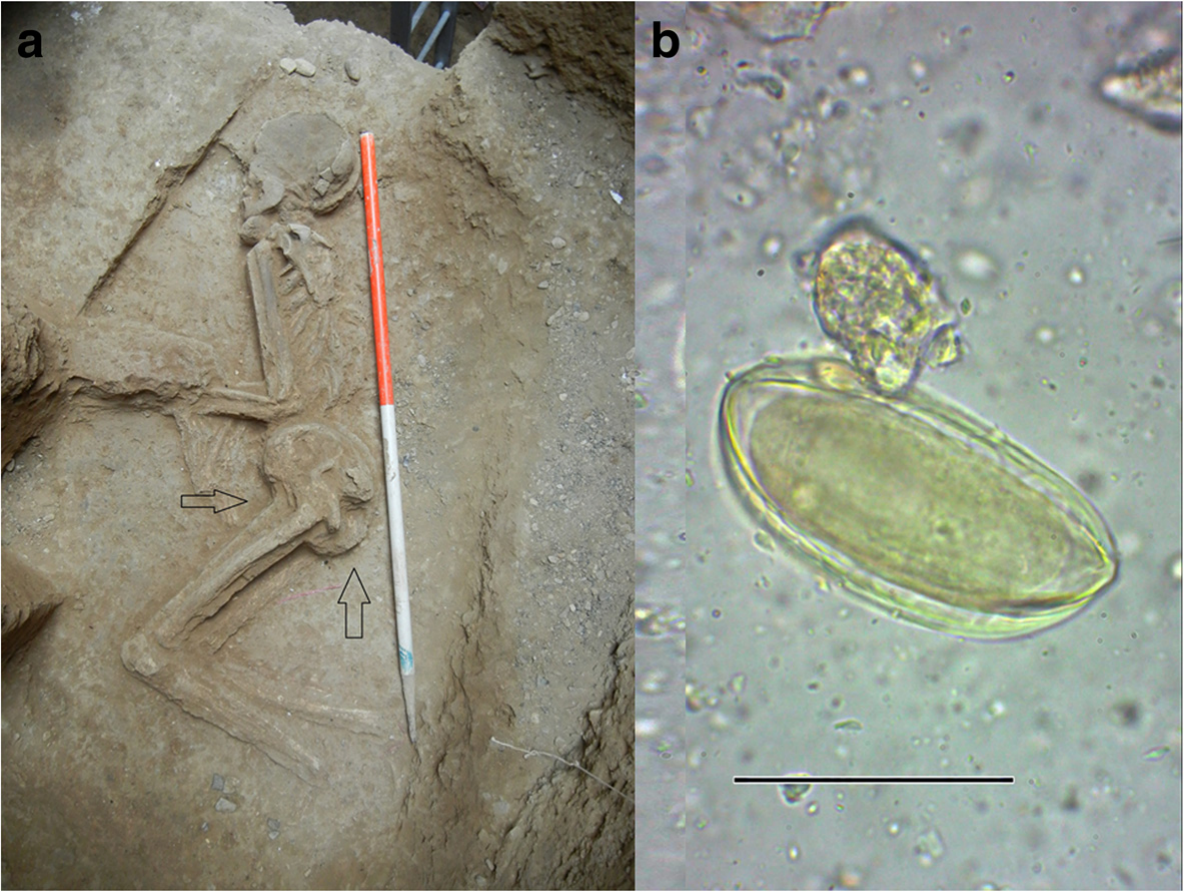
The entire skeleton of the female adolescent excavated in Tehran archeological site, (**a**) showing the points of sampling (**b**) *E.vermicularis* egg retrieved from the collected sample (bar: 50 μm)

The Molavi Street archeological site is located in the south of Tehran near the city’s Grand Bazaar. The site became widely highlighted in newspapers and news broadcasts when, in 2014, a piece of ancient pottery was found in soil close to a civil water and sewage construction project. Subsequent excavations, revealed a skeleton found in a fetal position, four meters under the street surface. Thermoluminescence dating of pottery found nearby the skeleton provided two dates: 6870 ± 300 and 6950 ± 280 BP [7]. Around 20 g of soil from the iliac fossa of pelvic bones, the coccyx, and apex regions of the sacrum were collected for paleoparasitological studies (Fig. 1a). For analysis, 10 g of the sample were rehydrated with TSP solution, as previously described [6, 8]. From the entire rehydrated specimen 488 microscopic slides were mounted and examined [9].

### Ethical Approval

Authors declare that the procedures of sampling, studying and reporting the current finding have followed in accordance with the ethical standards.

## Results & Discussion

Out of 488 microscopic slides that were examined only one egg similar to those of oxyurid worms was detected. At a first glance the apparent morphological features of the egg were undoubtedly those of *E. vermicularis*: the asymmetrical shape and the size of 30 × 60 μm that was within the usual size range (50 to 60 μm by 20 to 30 μm), as reported [9, 10]. Few smaller microscopic objects similar to cysts of protozoa and/or pollens were also observed in the rehydrated material. In the absence of reliable diagnostic methods all unidentified particles were preserved for further studies using molecular techniques. These findings from excavations at the Molavi street site have now extended the history of human residence in Tehran back to the 5^th^ millennium BC, whereas previous findings in Gheytarieh Cemetery in the northeast of Tehran estimated human presence to only 1000 years BC.

Identification of parasites in human and animal paleofeces obtained from the archeological sites has shed light on environmental conditions, levels of hygiene and many other habits of humans from the past [11]. *E. vermicularis* is a directly transmitted parasitic nematode provoking anal or perianal pruritus in young children. Air borne transmission of *E.vermicularis* that is well tolerated in communities nowadays [12] has been assumed to have developed in the distant past, when humans tended to settle in caves with limited air flow compared to their wandering ancestors who lived in open air environment [13]. The oldest record of a human infection is dated around 7837 BC in western Utah [14]. Several studies have also highlighted the controversial cause of appendicitis due to *E. vermicularis* and its documented role in female urogenital tract disorders that should be taken into account by surgeons, or gynecologists performing laparoscopy [3, 15]. According to paleoparasitological findings of *E.vermicularis* eggs from different parts of the world [16–18], the prevalence of infection seems to be higher in primitive populations of Neolithic period, being lower in personal and environmental hygiene level, than today's societies. Reports from different parts of the modern world [14, 19, 20], emphasize that this worm is the most prevalent intestinal helminthic infection. As female *Enterobius* releases her eggs around the perianal skin, stool examination is helpful in less than 5% of positive cases compared to the reference Graham's scotch test [21]. In conclusion, human paleofeces would not be appropriate samples for tracing enterobiasis in ancient humans, except in heavy infections such as the above mentioned oldest human enterobiasis record in Utah. However, due to more abundant parasite eggs near the anal region, the precise collection of soil samples from the sacral foramina and around the coccyx bones (Co1-Co4) could increase the chances of finding Oxyurid eggs (Fig. 1b). Distal phalanxes of fingers, mostly the index, could also be another place for sampling, as *Enterobius* eggs have been found beneath the nails in 60 % of infected cases reported in 1962 in Montreal, Canada [22]. In the present study, such samples could not be obtained, because of technical difficulties accessing the fingers and due to restrictions of official regulations. In addition, the clinical consequences of ectopic enterobiasis, particularly appendicitis [23, 24], generalized peritonitis [25], and female urogenital disorders [26] might have seriously threatened the life of primitive families. Therefore, the impact of enterobiasis on females and the well-being of children in pre-historic times should be re-evaluated throughout human history. The present parasitological and archeological finding at this burial has not only demonstrated the oldest possible occurrence of a human pinworm infection in this part of the world, but also has confirmed the human residency in the 5^th^ millennia BC in Tehran.

## References

[1] Mowlavi G, Kacki S, Dupouy-Camet J, Mobedi I, Makki M, Harandi MF, et al. Probable hepatic capillariosis and hydatidosis in an adolescent from the late Roman period buried in Amiens (France). Parasite. 2014;21. 10.1051/parasite/2014010 PMC3936287 24572211

[2] Iñiguez AM, Reinhard KJ, Araújo A, Ferreira LF, Vicente ACP (2003). Enterobius vermicularis: ancient DNA from North and South American human coprolites. Mem Inst Oswaldo Cruz.

[3] Ariyarathenam A, Nachimuthu S, Tang T, Courtney E, Harris S, Harris A (2010). Enterobius vermicularis infestation of the appendix and management at the time of laparoscopic appendicectomy: Case series and literature review. Int J Surg.

[4] Gonçalves MLC, Araújo A, Ferreira LF (2003). Human intestinal parasites in the past: new findings and a review. Mem Inst Oswaldo Cruz.

[5] Nezamabadi M, Aali A, Stöllner T, Mashkour M, Le Bailly M (2013). Paleoparasitological analysis of samples from the Chehrabad salt mine (Northwestern Iran). International Journal of Paleopathology.

[6] Mowlavi G, Makki M, Mobedi I, Araujo A, Aali A, Stollner T (2014). Paleoparasitological Findings from Rodent Coprolites Dated At 500 CE Sassanid Era in Archeological Site of Chehrabad (Douzlakh), Salt Mine Northwestern Iran. Iranian journal of parasitology.

[7] Zimmerman D (1971). Thermoluminescent dating using fine grains from pottery*. Archaeometry.

[8] Fugassa MH, Beltrame MO, Sardella NH, Civalero MT, Aschero C (2010). Paleoparasitological results from coprolites dated at the Pleistocene–Holocene transition as source of paleoecological evidence in Patagonia. J Archaeol Sci.

[9] Meyers WM, Neafie RC, Marty AM, Wear DJ. Pathology of infectious diseases. Volume 1: helminthiases. Washington, DC: Armed Forces Institute of Pathology, American Registry of Pathology, Washington, DC; 2000.

[10] Noble ER, Noble GA. Parasitology. The Biology of Animal Parasites. Academic Medicine 1964;39(9):872.

[11] Samuels R. Parasitological study of long-dried fecal samples. Memoirs of the Society for American Archaeology. 1965;175–179.

[12] Burkhart CN, Burkhart CG (2005). Assessment of frequency, transmission, and genitourinary complications of enterobiasis (pinworms). Int J Dermatol.

[13] Hugot J-P, Reinhard KJ, Gardner SL, Morand S (1999). Human enterobiasis in evolution: origin, specificity and transmission. Parasite.

[14] Cook GC (1994). Enterobius vermicularis infection. Gut.

[15] Tandan T, Pollard A, Money D, Scheifele D (2002). Pelvic inflammatory disease associated with Enterobius vermicularis. Arch Dis Child.

[16] de Araújo A, Ferreira LF, Confalonieri U, Nuñez L, Ribeiro Filho B (1985). The finding of Enterobius vermicularis eggs in pre-Columbian human coprolites. Mem Inst Oswaldo Cruz.

[17] Fry GF, Moore JG. Enterobius vermicularis: 10,000-year-old human infection. Science. 1969;166(3913):1620–0. 10.1126/science.166.3913.1620 4900959

[18] Araújo A, Ferreira LF (2000). Paleoparasitology and the antiquity of human host-parasite relationships. Mem Inst Oswaldo Cruz.

[19] Wang L-C, Hwang K-P, Chen E-R (2010). Enterobius vermicularis infection in schoolchildren: a large-scale survey 6 years after a population-based control. Epidemiol Infect.

[20] Park J-H, Han E-T, Kim W-H, Shin E-H, Guk S-M, Kim J-L (2005). A survey of Enterobius vermicularis infection among children on western and southern coastal islands of the Republic of Korea. Korean J Parasitol.

[21] Caldwell J (1982). Pinworms (enterobius vermicularis). Can Fam Physician.

[22] Royer A, Berdnikoff K (1962). Pinworm infestation in children: the problem and its treatment. Can Med Assoc J.

[23] da Silva DF, da Silva RJ, da Silva MG, Sartorelli AC, Rodrigues MAM (2007). Parasitic infection of the appendix as a cause of acute appendicitis. Parasitol Res.

[24] Mowlavi G, Massoud J, Mobedi I, Rezaian M, Mohammadi SS, Mostoufi N (2004). Enterobius vermicularis: a controversial cause of appendicitis. Iranian Journal of Public Health.

[25] Khan J, Steele R, Stewart D (1981). Enterobius vermicularis infestation of the female genital tract causing generalised peritonitis case report. BJOG: An International Journal of Obstetrics & Gynaecology.

[26] McKay T. Enterobius vermicularis infection causing endometritis and persistent vaginal discharge in three siblings. N Z Med J. 1989;102(861):56–6. 2739981

